# Assessing the reproducibility of exome copy number variations predictions

**DOI:** 10.1186/s13073-016-0336-6

**Published:** 2016-08-08

**Authors:** Celine S. Hong, Larry N. Singh, James C. Mullikin, Leslie G. Biesecker

**Affiliations:** 1 Medical Genomics and Metabolic Genetics Branch, National Human Genome Research Institute, National Institutes of Health, Bethesda, MD 20892 USA; 2 NIH Intramural Sequencing Center, National Human Genome Research Institute, National Institutes of Health, Bethesda, MD 20852 USA; 3 Comparative Genomics Analysis Unit, Cancer Genetics and Comparative Genomics Branch, National Human Genome Research Institute, National Institutes of Health, Bethesda, MD 20852 USA

**Keywords:** Copy number variations (CNV), Exomes, CNV predictions, Reproducibility

## Abstract

**Background:**

Reproducibility is receiving increased attention across many domains of science and genomics is no exception. Efforts to identify copy number variations (CNVs) from exome sequence (ES) data have been increasing. Many algorithms have been published to discover CNVs from exomes and a major challenge is the reproducibility in other datasets. Here we test exome CNV calling reproducibility under three conditions: data generated by different sequencing centers; varying sample sizes; and varying capture methodology.

**Methods:**

Four CNV tools were tested: eXome Hidden Markov Model (XHMM), Copy Number Inference From Exome Reads (CoNIFER), EXCAVATOR, and Copy Number Analysis for Targeted Resequencing (CONTRA). To examine the reproducibility, we ran the callers on four datasets, varying sample sizes of *N* = 10, 30, 75, 100, 300, and data with different capture methodology. We examined the false negative (FN) calls and false positive (FP) calls for potential limitations of the CNV callers. The positive predictive value (PPV) was measured by checking the CNV call concordance against single nucleotide polymorphism array.

**Results:**

Using independently generated datasets, we examined the PPV for each dataset and observed wide range of PPVs. The PPV values were highly data dependent (*p* <0.001). For the sample sizes and capture method analyses, we tested the callers in triplicates. Both analyses resulted in wide ranges of PPVs, even for the same test. Interestingly, negative correlations between the PPV and the sample sizes were observed for CoNIFER (ρ = –0.80). Further examination of FN calls showed that 44 % of these were missed by all callers and were attributed to the CNV size (46 % spanned ≤3 exons). Overlap of the FP calls showed that FPs were unique to each caller, indicative of algorithm dependency.

**Conclusions:**

Our results demonstrate that further improvements in CNV callers are necessary to improve reproducibility and to include wider spectrum of CNVs (including the small CNVs). These CNV callers should be evaluated on multiple independent, heterogeneously generated datasets of varying size to increase robustness and utility. These approaches to the evaluation of exome CNV are essential to support wide utility and applicability of CNV discovery in exome studies.

**Electronic supplementary material:**

The online version of this article (doi:10.1186/s13073-016-0336-6) contains supplementary material, which is available to authorized users.

## Background

Reproducibility is receiving increasing attention across the biomedical enterprise [1]. Genomics is no more or less affected by issues of reproducibility, but because it is a fast-moving field, there have been few opportunities to evaluate reproducibility. With advances in next-generation sequencing (NGS) technology, new tools are constantly being developed to mine and maximize the utility of NGS data. A major challenge with computational predictions is the reproducibility of the performance on a dataset other than the one it was trained on. A common mistake is overfitting, wherein the performance of the prediction is adjusted for an optimal outcome under a narrow set of conditions, which may not be reproducible in other datasets.

In this study, we examined the reproducibility of copy number variation (CNV) predictions from exome sequence (ES) data. While studying CNVs is not the primary purpose of ES, by applying computational methods, it may be possible to predict putative CNVs from ES data. In fact, integrating substitutions and indel mutation results from ES data with CNV data would increase the power of ES studies. To this end, numerous tools are now available to predict CNVs from ES data, and more are continuing to be developed [2–18].

Unlike genome sequence (GS) data, in addition to the problems arising from GC contents and low complexity regions, the targeted enrichment ES methodology produces non-uniform read depths, which can confound CNV predictions. Despite the challenges, the relatively lower cost of ES compared to GS coupled with the limited current capability to interpret the non-protein-coding regions in a genome makes ES an attractive option. CNVs are becoming increasingly important, because about 9 % of mutations that cause Mendelian diseases can be considered as CNVs [19]. Thus, accurate exome CNV prediction would be a valuable resource. Discovering CNVs from ES data is a challenging and complicated problem. This is reflected by the proliferation of tools and the lack of consensus in the field on the best computational method. While a handful of CNV caller comparison papers have been published, no study has investigated the reproducibility of CNV prediction to the best of our knowledge [20–22].

In this study, we set out to examine the performance reproducibility of population-based CNV callers from exome data. We included population-based callers that were publicly available when this study began. The callers included in this study were: CoNIFER (Copy Number Inference From Exome Reads), CONTRA (Copy Number Targeted Resequencing Analysis), EXCAVATOR, and XHMM (eXome-Hidden Markov Model) [4, 9, 10, 14]. These callers have different approaches to pre-processing the data and predicting CNVs, as summarized in Table 1. By testing heterogeneous experimental conditions, we objectively evaluated the reproducibility of these methods.

**Table 1:** Summary of methods used in CNV callers

CNV caller	Pre-processing quality control	Approach to discovering CNVs	Published validation rate
CoNIFER [9]	RPKM for each target (filter targets with median RPKM <1), ZRPKM , SVD-PCA transformation. Filter samples >0.5 SVD-ZRPKM	±1.5 SVD-ZRPKM threshold values	94 % PPV
CONTRA [4]	Removes base coverage <10, library-size correction by removing linear dependency between log-coverage and log-ratio	Base-level log-ratios using adjusted coverage, followed by region-level log-ratios using mean of base-level log ratios. Two-tailed *p* value on normal distribution of region-level log-ratio. Heuristic approach of using different solutions of segmentations for large CNVs	86.8 % SPE 95.4 % SEN
EXCAVATOR [14]	Data correction by using the medians of Exon-mean-read-count values respect to GC content, mappability, and exon sizes. Log-transformed ratio, LOWESS scatter plot normalization	HMM to discover five states of CNVs (double loss, loss, neutral, gain, or multiple gain)	~50 % PPV
XHMM [10]	Filter extreme GC content (<0.1 or >0.9), low complexity (>10 %), target size (<10 bp or >10 kb), samples (mean RD <25 or >500), targets (Mean RD <10 or >500). SVD-PCA normalization, remove K components = 0.7/n s	Z-score calculation as input for three-state HMM	67–92 % SEN

We tested the callers under three conditions: varied data input (i.e. data generated from several sequencing centers), varied sample sizes, and distinct capture methodology. We obtained the data generated by the NIH (ClinSeq*®*), Broad Institute (BI), and the Washington University Genome Sequencing Center (WUGSC). We included 971 samples from ClinSeq*®*, 167 samples from the BI, 116 samples from the WUGSC (see “Methods”). All three ES datasets were ascertained from primarily healthy people. This criterion was important in our study, as patient populations can have higher number of structural variants, including CNVs [23].

Since the data from different centers can have many confounding variables, to further test the reproducibility with minimized confounders, we ran the CNV callers on subsets of ClinSeq*®* data. By using ClinSeq*®*, we minimized the sequencing center effect and other variability that may rise from downstream analyses. To test the reproducibility for varying sample sizes, we randomly sampled from a pool of ClinSeq*®* samples that were captured and sequenced with the same protocol and processed with the same bioinformatics pipeline. Lastly, we also tested the reproducibility of CNV prediction reliability on different capture methodology. The sample size and capture methodology analyses were tested in triplicates by random sampling, allowing us to examine the reproducibility for the same condition runs.

## Methods

### Data collection

Three datasets were collected to objectively test the performance of CNV predicting programs. To minimize the sequencing center bias, the ES data from BI and WUGSC from the 1000 Genome Project were downloaded. Only the samples with CNV experimental data available were included in this study. A total of 168 ES data from BI and 116 ES data from WUGSC were included (see the Additional file 1: Table S1). The corresponding CNV data (Affy 6.0 and Illumina 1 M arrays) were obtained from the Hapmap Project website (fPPV://fPPV.ncbi.nlm.nih.gov/hapmap/cnv_data/). The Hapmap CNV data were lifted over to hg19 using UCSC Liftover tool. ClinSeq® was used as an independent source of ES data (dbGaP Accession phs000971.v1.p1). In total, 971 ES samples were included. The ClinSeq® samples were captured by one of the following methods: Agilent SureSelect Human Exon (38 MB), Agilent SureSelect ICGC (50 Mb), Illumina TruSeq v1,v2. All samples were sequenced by the NIH Intramural Sequencing Center (NISC) and aligned to hg19 reference genome. Due to the heterogeneous capturing methods used for samples, only the intersecting targets were used in this study. The IDs for samples included in this study are listed in the Additional file 1: Table S1.

### Exome CNV predicting programs

Four CNV predicting software packages were downloaded and installed: XHMM, CoNIFER, CONTRA, EXCAVATOR [4, 9, 10, 14]. XHMM, CoNIFER (v-0.2.2), EXCAVATOR Package (v2.2), and CONTRA (v2.0.4). CoNIFER was run on python version 2.7.5 with pysam version 0.6. For CONTRA, python v2.6, R version 2.13.0, and bedtools version 2.16.2 were used. XHMM was run with GATK version 3.1-1 and plinkseq version 0.09. The QC analysis for data was followed as recommended by each CNV tool and the default parameters were used. The software was run as is and no custom scripts were added to the original codes. For CoNIFER, we set the number of singular values to remove as 6, a liberal value, to include any copy number polymorphisms discovery. The scree plots were examined and this number was near the inflection point for all runs. Table 2 summarizes different tests run on each CNV callers. The association analyses were performed using R. The ANOVA and the Spearman’s rank correlation were calculated.

**Table 2:** Summary of CNV runs on different callers

Dataset	XHMM	CoNIFER	CONTRA	EXCAVATOR
BI (167)	O^a^	O	–^b^	O
WUGSC (116)	O	O	–	O
BI GIH (48)	O	O	O	O
ClinSeq® (54)	O	O	O	O
Sample size analysis (ClinSeq® in triplicates by random sampling)				
10	O	O	–	O
30	O	O	–	O
75	O	O	–	O
100	O	O	–	O
300	O	O	–	O
Capture kit analysis (48 samples from ClinSeq® in triplicates by random sampling)				
SS HAE	O	O	–	O
SS ICGC	O	O	–	O
TruSeq v2	O	O	–	O
Mix capture^c^	O	O	–	O

*BI* Broad Institute, *WUGSC* Washington University Genome Sequencing Center, *BI-GIH* Broad Institute Gujarati Indians in Houston, Texas

^a^O denotes that a caller was run on a given dataset

^b^– denotes that a caller was not run on a given dataset

^c^Indicates data comprising 48 samples, 16 samples from each of the three capture kit samples

### Analyses datasets

For varied data input, we used four datasets: 54 ClinSeq® samples, 116 WUGSC samples, 167 BI samples, and 48 BI Gujarati Indians (BI GIH) samples. For different sample size analysis, sample sizes of n = 10, 30, 50, 75, or 300 were chosen from ClinSeq®. For each sample size, the runs were performed in triplicates by random sampling, except for sample size 300. For sample size 300, duplicate experiments were performed. To minimize the capture kit bias, the samples were selected from Agilent SureSelect Human All Exon capture kits. For capture kit analysis, four datasets were used from ClinSeq®: (1) exomes captured by Agilent SureSelect Human All Exon capture kit; (2) Agilent SureSelect ICGC capture kit; (3) Illumina TruSeq v2 capture kit; (4) heterogeneous mixture of exomes from all three capture enrichment kits. Mixed capture data were composed of 16 samples randomly selected from each of the three capture kits, totaling 48 samples. Forty-eight samples were randomly drawn three times for all datasets. Plots were generated using ggplot2 R library package.

### Assessing reproducibility

For the 1000 Genomes Project samples, available CNV data were used to check the accuracy of calls (see “Data collection” section). For the ClinSeq*®* set, we used a single nucleotide polymorphism (SNP) array to validate the CNVs. All samples were run and analyzed by the NHGRI Genomics Core, NIH. A total of 496 samples were run either on Illumina Infinium HumanExome-12 v1.1 BeadChip, Illumina Infinium HumanExome-12 v1.2 BeadChip, or Illumina Infinium Immuno BeadChip. Samples with a call rate of >0.99 were considered and SNPs with GenTrain score <0.7 were filtered out. CNVPartition v3.2 (minimum contiguous SNPs = 4, confidence threshold = 0, GC wave adjust = True) and Nexus v7.5 (minimum contiguous SNPs = 4, significance threshold = 5E-7, maximum contiguous SNP spacing (kbp) = 1000) were used to call CNVs. We took an intersection of the regions between the CNV experimental data and exome target location. The predicted CNVs were considered as subject to validation if the predicted regions span at least four SNPs from chip data. Bedtools version 2.19.0 was used to intersect genomic regions. Positive predicted value (PPV) was defined as CNV calls supported by SNP array out of all predicted CNVs that are subject to validation, while false positive (FP) was defined as CNV calls not supported by the SNP array out of all predicted CNVs that are subject to validation. Sensitivity was defined by the number of CNVs called by both CNV predictions and SNP array, out of the total known SNP array CNVs in the dataset that have at least one exome target. Chromosomes Y and X were removed from this study.

## Results

### Reproducibility in varying data input

Sequencing centers need to be able to select a caller that will make robust predictions of CNVs. Unfortunately, most CNV callers are developed and piloted on only a single or a limited dataset. We set out to examine the performance of several callers on heterogeneous datasets to better understand the potential real-world behavior of the programs. We ascertained ES data from three independent centers (ClinSeq*®,* BI, WUGSC) and examined the variability of four prediction traits: the number of CNV calls per sample, predicted CNV sizes, the call type percentages (duplications/deletions), and PPV. We chose the PPV as the standard calculation throughout this study to measure the validity of the calls, as the sensitivity alone does not accurately reflect the reliability of CNV calls and can be driven by high or low numbers of calls. In addition, it is more valuable to know how likely the calls are true, in practice, when applying CNV callers to identify putative CNVs for further validation. The PPV was defined as $\frac{true\; CNVs}{all\; CNVs\; predicted}$. True CNVs were defined as the number of CNV calls that were concordant between the exome CNV prediction and the experimental data (see “Methods”).

In these analyses, four datasets were used. We included 167 samples from BI, 116 samples from WUGSC, 48 samples of BI Gujarati Indians descent, and 54 ClinSeq*®* samples. The BI Gujarati Indians samples were isolated as one dataset from the larger BI samples and 54 ClinSeq*®* samples were randomly selected from the whole ClinSeq*®* cohort (see “Methods”). All callers were run using default settings. We were only able to run CONTRA on smaller datasets as additional source code modification was required to run CONTRA on larger sample datasets. While we did not run CONTRA on large datasets, we still show our results obtained from running CONTRA on smaller sets, as the purpose of this study is not to compare the callers but to examine the reproducibility. All runs are summarized in Table 2. For each run, we examined the following traits: CNV calls per sample, CNV size, percentage of call types (duplication, deletion), and PPV. The association between each trait versus data input was calculated using R Analysis of Variance (ANOVA) package.

**Fig. 1:**
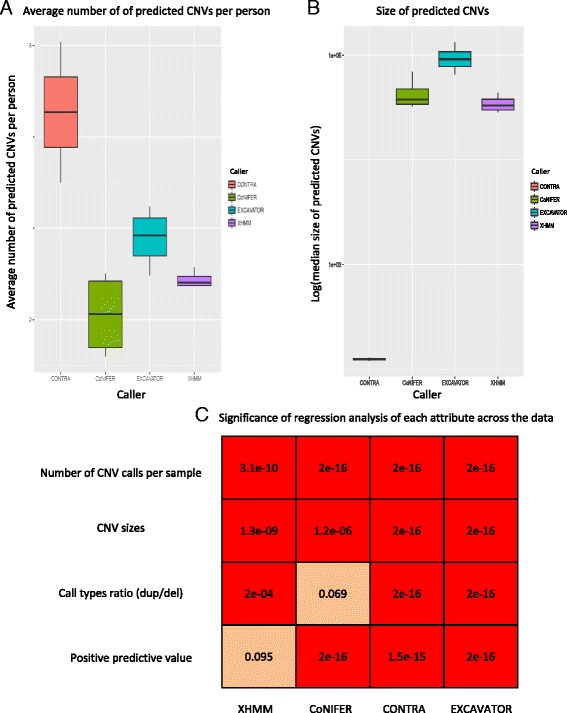
Examining the number of CNVs called, sizes, and correlations. **a** The *boxplot* of the number of average number of CNVs per sample across the dataset **b** The significance of association between the exome study attributes (*X-axis*) and varying data input. Each *row* shows association values for the given caller (*Y-axis*). Reliability = PPV. **c** *Boxplots* for predicted CNV median size distribution

Because these samples were from primarily apparently healthy individuals, we hypothesized that the mean number of CNV calls per sample $\left(\overline{X}\right)$ should be similar. For each caller, we examined the average number of CNV calls per sample $\left(\overline{X}\right)$ for each run, where $\overline{X}=\frac{Total\; Number\; of\; CNVs\; called}{Number\; of\; samples}$. We calculated standard deviations (SD) to examine the dispersion of $\overline{X}$ between the runs for each caller to examine the difference in the number of calls made between each simulated experiments. The $\overline{X}$ for EXCAVATOR was in the range of 20–88 (SD = 30). The $\overline{X}$ was in the range of 3–20 (SD = 10.8) and 15–23 (SD = 3.6) for CoNIFER and XHMM, respectively (Fig. 1a). The $\overline{X}$ range for CONTRA was 148–3241 (SD = 2187). The $\overline{X}$ varied significantly for each dataset for all callers (*p* <0.001, see Fig. 1b).

**Fig. 2:**
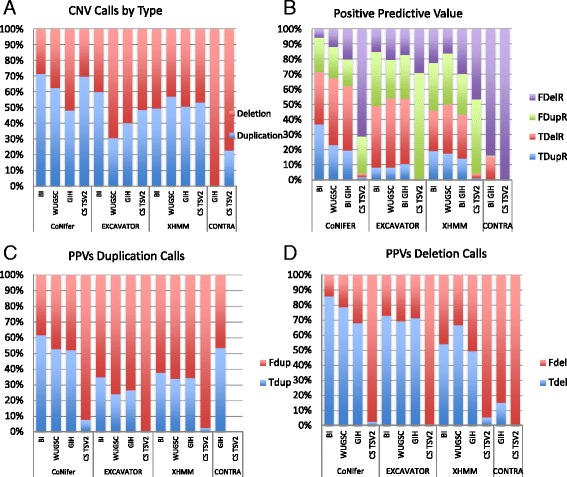
Examining CNV calls by type. **a** Percentages of deletion and duplication calls. **b** PPVs categorized by duplications and deletions. **c** PPVs of duplication calls. **d** PPVs of deletion calls. *FdelR(Fdel)* deletion calls that were not verified, *FdupR(Fdup)* duplication calls that were not verified, *TDelR(Tdel)* deletion calls that were verified, *TdupR(Tdup)* duplication calls that were verified

Next, we examined the median sizes and call types of the CNV calls across the datasets for each caller. The median sizes of CONTRA calls were in the range of 121–129 bp (SD = 5.7 bp). The median sizes were in the range of 32–69 kb (SD = 17 kb) for CoNIFER, 7.5–36 kb (SD = 12 kb) for XHMM, and 65–133 kb (SD = 29 kb) for EXCAVATOR (Fig. 1c). For all callers, CNV sizes were associated with data input (*p* <0.001, Fig. 1b). For call types, we examined the percentages of each call type (duplications or deletions). Deletion calls were in the range of 70–99 % for CONTRA, 30–52 % deletions for CoNIFER, 40–69 % for EXCAVATOR, and 43–51 % for XHMM (Fig. 2a). The percentages of each call types were variable among datasets for CONTRA, EXCAVATOR, and XHMM (*p* <0.001, Fig. 2).

The reliability of the CNV calls was measured by the PPV (see “Methods” and Additional file 1: Table S2 for more information). We calculated the PPV for duplication and deletion calls separately. For duplication calls, the ranges of PPV were 0–0.53 for CONTRA, 0.07–0.61 for CoNIFER, 0–0.31 for EXCAVATOR, and 0.20–0.37 for XHMM. For deletion calls, the range of PPV was 0–0.15 for deletions and 0–0.53 for duplications (Fig. 2a–d). For CoNIFER, the range of PPV was 0.07–0.61. All callers had higher PPVs for deletions (Fig. 2c, d). The PPVs for deletions were in the range of 0–0.15 for CONTRA, 0.02–0.86 for CoNIFER, 0–0.73 for EXCAVATOR, and 0.05–0.67 for XHMM. PPV observed for all calls was significantly variable between each run for CoNIFER, EXCAVATOR, and CONTRA (*p* <0.001, Fig. 2).

In summary, all four attributes of CNV calls examined in this study (the number of CNV calls per sample, median sizes, the percentages of call types, and the PPV) significantly varied between each run (*p* <0.05). Dispersion observed using SD showed a wide range of values for these traits. The results from our analyses showed that the CNV predictions from distinct exome dataset yield dataset-specific number of CNV calls per sample, median sizes, the percentages of call types, and the PPV, mediated by the confounders of exome methodology.

### Effects of sample size

Data collected from different sequencing center can have multiple confounders. Therefore, we further tested the reproducibility of CNV callers using different sample sizes with minimized confounding effects. We simulated a series of experiment sizes by analyzing subsets of ClinSeq*®* data. To minimize confounding effects, we eliminated the heterogeneity of capture kit bias by using only the Agilent SureSelect Human Exon (38 Mb) capture data. We selected sample sizes (n) of 10, 30, 75, 100, and 300 exomes and applied CoNIFER, EXCAVATOR, and XHMM to predict CNVs. CONTRA was excluded in this analysis for the same reason mentioned in the previous section. For each set, the samples were randomly chosen in separate, triplicate runs for each size. We examined the correlation of the PPV and the sample sizes by calculating the Spearman’s rank correlation coefficients (ρ) for all callers. Fewer than 10 CNVs subject to validation were detected for sample sizes 10 using XHMM and CoNIFER (see Additional file 1: Table S3), thus the results from sample size 10 were excluded.

**Fig. 3:**
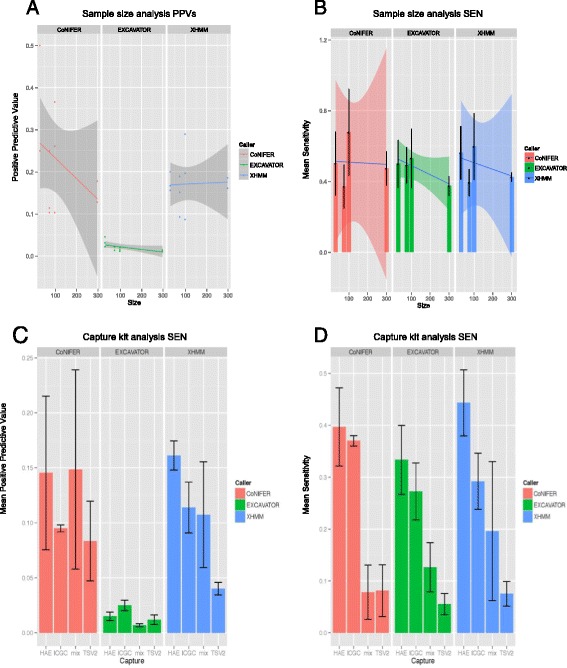
PPV and SEN for triplicate runs for sample size and capture kit analysis. **a** PPVs for sample size analysis. Each *dot* represents a single run. **b** SEN for sample size analysis. **c** PPV for capture kit analysis. **d** SEN for capture kit analysis. The mean and the standard error for the triplicate runs are graphed for B–D. *HAE* SureSelect HAE kit, *ICGC* SureSelect ICGC capture kit, *mix* simulated mixed capture kit data, *TSV2* Illumina TruSeq v2 capture kit

Surprisingly, the PPVs of CoNIFER (ρ = –0.80) and EXCAVATOR (ρ = –0.77) calls were strongly and negatively correlated with the sample sizes. CoNIFER had the highest PPV at sample size of 30 (Fig. 3a). Based on a negative correlation between the PPV and the sample sizes, in conjunction with the increasing number of CNV calls per sample, we concluded that the CoNIFER calls were less reliable as the sample sizes increased. Even though a negative correlation was observed for EXCAVATOR, no valid conclusions could be made as the PPVs were consistently low (<0.05) for all sample sizes No correlation of sample size and PPV was observed for XHMM (Fig. 3a).

Interestingly, we observed a wide range of PPV even within replicate sample size runs, some by more than threefold differences. For example, for a sample size of 100, the PPV was in the range of 0.10–0.36 for CoNIFER and 0.09–0.29 for XHMM. EXCAVATOR had PPV <0.05 for all runs. However, EXCAVATOR had sensitivity >0.80, presumably being driven by the high number of CNV calls (Fig. 3a, b and Additional file 1: Table S4, S5).

The observed results show the lack of reproducibility in PPV. The published performance of CNV callers could not be replicated in this analysis (Table 1). The correlation of the sample size and the PPV may be explained by noise introduced by additional samples that are not successfully removed and normalized. The range of PPV values for the replicate runs demonstrated the inconsistency of PPV. Based on this, we concluded that the exome CNV calling reproducibility is a challenge even with minimized confounders.

### Effects of capture enrichment kit heterogeneity

Lastly, we examined the reproducibility of CNV performance using a distinct capture kit data generated at a single sequencing center. We used ClinSeq® data comprising three capturing enrichment kits: Agilent SureSelect Human All Exon 38 Mb (HAE), Agilent SureSelect ICGC 50 Mb (ICGC), and Illumina TruSeq V2 (TSV2). Four datasets were used: HAE, ICGC, TSV2, and heterogeneous capture (mix). The simulated single capture data (HAE, ICGC, TSV2) consisted of 48 samples randomly chosen from a sample pool of each capturing method. The mix set consisted of 16 randomly chosen samples from each of the three capturing methods. This was repeated three times.

First, we tested the significance of relationships between each capturing method and the PPVs for all calls. We hypothesized that the single capture data (HAE, ICGC, or TSV2) would have higher PPV than the heterogeneous capture data (mix). To test this, we checked for association of PPV and the data category (single or mix capture) using linear model association. No significant association was observed (*p* >0.05). We then tested the association of PPVs and each capture methodology (HAE, ICGC, TSV2, mix). No significant association was observed (*p* >0.05). This was explained by the wide range of PPVs observed for all callers, as was seen in the sample size analyses (Fig. 3c, d and Additional file 1: Table S4). For example, the range for mix sample was 0–0.31 for CoNIFER, 0.05–0.20 for XHMM, and 0–0.01 for EXCAVATOR. As seen previously, we observed PPV <0.05 for all EXCAVATOR runs (Fig. 3c). Sensitivity was comparable to other callers, again, driven by high number of CNV calls (Fig. 3d). We did not observe the high prediction accuracy as in published results for the callers (Table 1). In addition, triplicate runs sampling from the same sample pools showed a wide range of PPV values. We concluded that none of the CNV callers we have evaluated here perform reliably across heterogeneous sample sets.

### Examining CNVs at exon-resolution

**Fig. 4:**
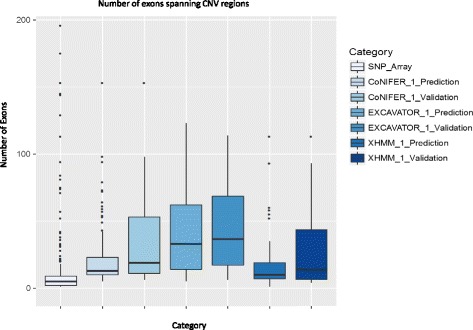
*Boxplots* of number of exons spanning the CNV regions. *X-axis* category for each boxplot, *Y-axis* the number of exons spanning CNVs

Using a 300 ClinSeq*®* samples, the largest sample size in this study, we examined the CNVs using exon-resolution to better understand the potential limitations of the callers. The CNV sizes from the SNP array were significantly smaller compared to the computationally predicted CNVs (Fig. 4). The median number of exons spanning CNVs for SNP array was 5, compared to 13, 33, and 10 for CoNIFER, EXCAVATOR, and XHMM, respectively (see Additional file 1: Table S6). No CNVs with <5 exons were predicted using CoNIFER and EXCAVATOR. Interestingly, the median number of exons for true positives (TP) was higher than the median number of exons for all predicted CNVs (TP + FP) (Fig. 4, Additional file 1: Table S6, and Additional file 2: Figure S1).

Of the known CNVs in the 300 ClinSeq*®* cohort, 55 % of the CNVs were not detected by all callers (false negative calls (FN)). These FN calls were heavily biased towards the smaller CNVs; 46 % of the CNVs spanned ≤3 exons and 88 % of the CNVs spanned ≤8 exons (see Additional file 1: Table S7). This indicated limitations in detecting small CNVs from exome. Of the FN CNV calls, 24 % of the FN calls for CoNIFER, 22 % of EXCAVATOR calls, and 25 % of XHMM calls were accurately called as CNV by at least one other caller. This indicated that the RD CNV signals were present in the exome sequencing data and may have been mitigated through the normalization and/or did not reach the threshold in the discovery algorithm.

### Examining the false positive calls

Using a 300 ClinSeq*®* sample run, we examined the FP regions for the three callers to examine the overlap. More than 90 % of the FP calls were unique to a caller and only 14 FP were called by all three callers, which was 10 % of the FP calls for CoNIFER, 1 % for EXCAVATOR, and 13 % for XHMM (Additional file 2: Figure S2). This suggested that the majority of the false calls were unique to a caller. This indicated that the FP calls were attributable to different normalization and discovery algorithms.

## Discussion

Our study demonstrates the challenges of CNV predictions from exomes. The major issue we observed was the lack of reproducibility of PPV among distinct exome datasets. Even when the confounders of exome methodology were minimized (e.g., same sequencing center, same capture methodology, same sample size, same bioinformatics pipeline), the triplicate runs still yielded a wide range of PPVs, seen in both sample size and capture analyses. We concluded that the performance of CNV calling is data-dependent and could not be generalized. Our results indicated that each simulated experiment was unique and resulted in an inconsistent PPV. This also explains why other studies have failed to reproduce the same prediction performance [20, 21, 24].

There are many factors that can contribute to the complexity and uniqueness of exome data. A well-known contributor is the GC content. High or low GC content regions of genomes are difficult to sequence due to the reduced amplification in PCR steps and thus results in poor coverage [25]. In addition, as GC content affects the target affinity, it can affect the target hybridization efficiency and/or the enrichment [26]. Thus, even with the same capture enrichment method, the coverage from one sample to another for the same regions can vary significantly. While many CNV callers address the GC effect by normalizing or filtering out for extreme GC targets, successfully removing GC bias is still a challenge.

The pre-processing and normalization steps adjust for such biases occurring in exome data. This step is crucial to systematically remove noise stemming from the various steps of exome methodology, which otherwise gives rise to a FP CNV signals. Retaining a true CNV signal while removing noise is a challenging problem. Uniquely, EXCAVATOR corrects for GC content, mappability, and library size by using a median normalization approach, as opposed to the filtering methods [14]. While this normalizes the data, this approach is not able to remove problematic extreme regions and is thus susceptible to high FP predictions. This may explain the low PPVs observed this throughout this study (PPV <0.05). While both CoNIFER and XHMM use the SVD-PCA normalization method, the negative correlation with sample sizes was observed only for CoNIFER. This suggested that the difference in results is due to the CNV discovery step. CoNIFER uses SVD-ZRPKM cutoff to determine the CNV signals. A fixed threshold cutoff may not be appropriate for all sample sizes due to increase in the noise as more data are added. An adjustment for increased sample size may be appropriate for a large population study.

Our results indicated that a dataset with heterogeneous capture methodologies did not negatively affect the CNV calling compared with a capture data from a single methodologic source. This is surprising because we expected that the mix capture data would introduce false signals due to the difference in probe designs. However, that effect was not observed. We concluded that the biases arising from different capture designs were successfully removed and normalized.

We observed challenges with detecting small CNVs using all callers. CoNIFER requires at least three exons to exceed the pre-determined threshold whereas EXCAVATOR requires at least two. However, the smallest predicted CNV spanned five exons and no CNVs were detected at this pre-defined minimum number of exons. Further examination of the FP calls showed that they were unique to a caller. This indicated that the FP calls were driven by the normalization pre-processing and/or the discovery algorithm. An improvement in normalization and the discovery algorithm of CNVs is necessary to reduce the FP calls and to detect small CNVs.

In this study, we have used the recommended default parameters for all CNV callers. Optimizing and tuning the parameters may improve the CNV prediction performance. However, this requires prior knowledge of existing true CNVs in the dataset, which oftentimes is not available for researchers using exome CNV callers to screen for CNVs. In addition, tuning the parameters to optimize the performance of a subset of data may potentially lead to an over-fitting, contradicting the objective of this project. We also appreciate the limitations of SNP chips in accurately identifying CNVs [27]. This was one of the reasons why we decided to measure the concordance of the CNV calls from exome against the SNP chip data (PPV) rather than focusing on the sensitivity and specificity. While PPV is a subset of accuracy, it still gives an estimate on the quality of the CNV predictions and was appropriate for this study to examine reproducibility in performance.

Our results unequivocally demonstrate the challenges in reproducibility of exome CNV calling. One way to improve the reproducibility of CNV calling would be to train and test the algorithm on large number of datasets. For a classifier in a machine learning approach, one of the techniques to reduce the over-fitting the data and variance of the performance is to use *k-*fold cross-validation, where the suggested *k ≥* 10 [28]. Similar to this concept, the field would benefit from a large, standardized set of heterogeneously generated exome data upon which CNV callers could be validated and the optimal parameters can be tested on. This set should include a wide range of sample sizes, independent data that were generated from different sequencing centers, and different ethnic backgrounds. In addition, understanding the attributes of genes that lead to high or low PPVs could further improve on the algorithms of CNV callers. For example, genomic regions with similarity in sequence or (retro)pseudogenes are likely to give FFP CNV signals. Characterization of CNV frequency for a gene or a gene CNV tolerance level in conjunction with frequencies of CNV calls in a population may be insightful. Incorporating gene attributes in a model may enhance the ability to distinguish the true CNVs versus artifact signals arising from ambiguous mapping of genomic regions or noise. We also recommend evaluating for PPV, in conjunction with the sensitivity and specificity. We repeatedly observed high sensitivity with low PPV. Exome callers are typically applied when genome-wide CNV screening is not available. Thus, high PPV is desired in practice. An effort to detect small CNVs (≤3 exons) is also necessary to study a larger spectrum of CNVs and increase the utility of extracting CNVs from exome data.

## Conclusion

By examining the exome CNV predictions under heterogeneous conditions, we have shown that the reproducibility of four CNV caller predictions was poor. The technical confounders and complexity of ES methodology contributed to the uniqueness of individual datasets, which made the reproducibility of CNV prediction performance a challenge. While CNV callers are potentially valuable and could increase the power and utility of ES studies, we have shown that there is still a need for improvement. As more ES and complementing high-quality CNV studies become available and further improvements are made in CNV calling algorithms, the wide applicability of CNV callers will maximize the utility of ES data.

## Abbreviations

CNV, copy number variations; ES, exome sequencing; GS, genome sequencing; NGS, next-generation sequencing; PPV, positive predictive value

## Additional files

Additional file 1: Table S1. Lists all the IDs used in this study. **Table S2** gives details of independent dataset analysis. **Tables S3 and S4** give details of size and capture analyses. **Table S5** lists ClinSeq*®* CNVs identified from SNP chips used in this study. **Table S6** lists the summary of CNV sizes for 300 ClinSeq run at base pair and exon resolution. **Table S7** lists SNP array CNV calls used for 300 ClinSeq samples. (XLSX 57 kb) Additional file 2: Figure S1. Is the density plot of size comparison between the predicted CNVs and the validated CNVs, **Figure S2** shows a Venn diagram of false positives overlapping between the callers. (PPTX 289 kb)
