# Supplementary material for: Assessing the reproducibility of exome copy number variations predictions
Source: Genome Med. 2016 Aug 8;8:82. doi: 10.1186/s13073-016-0336-6 (PMC4976506; doi:10.1186/s13073-016-0336-6)
Supplement: Additional file 2: Figure S1. — Is the density plot of size comparison between the predicted CNVs and the validated CNVs, Figure S2 shows a Venn diagram of false positives overlapping between the callers. (PPTX 289 kb) [file 13073_2016_336_MOESM2_ESM.pptx]

## Slide 1
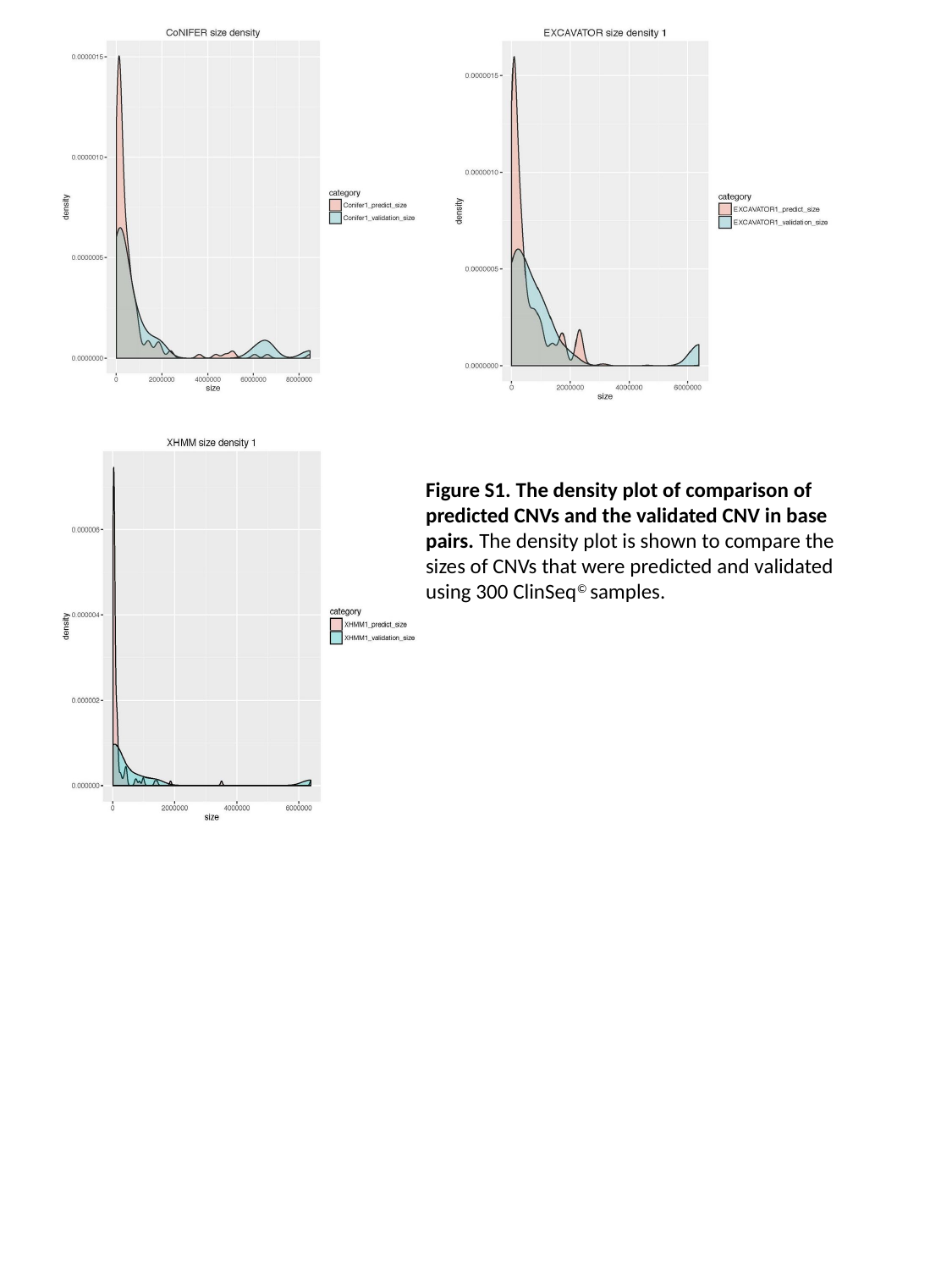

Figure S1. The density plot of comparison of predicted CNVs and the validated CNV in base pairs. The density plot is shown to compare the sizes of CNVs that were predicted and validated using 300 ClinSeq© samples.

## Slide 2
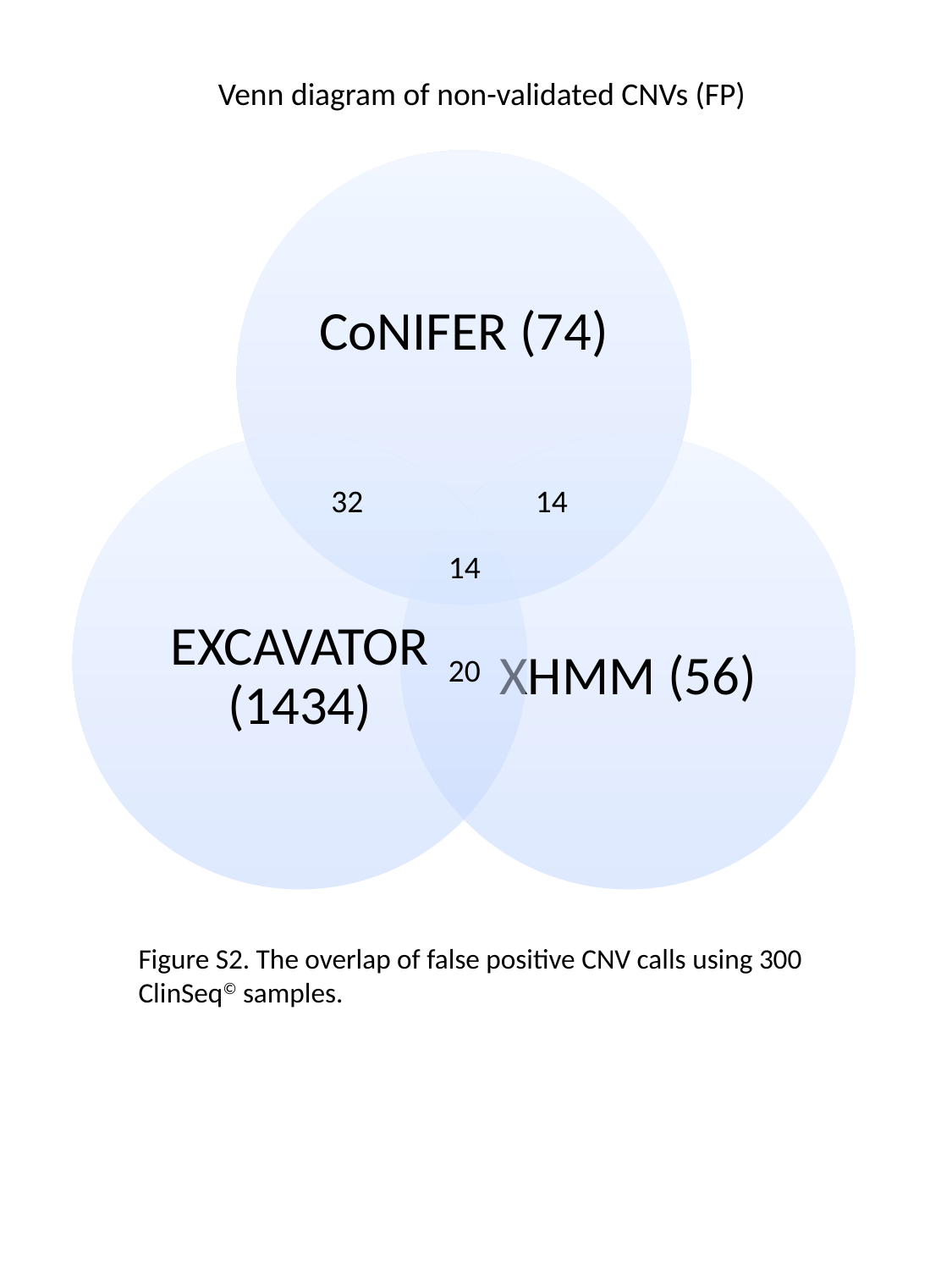

Venn diagram of non-validated CNVs (FP)
32
14
14
20
Figure S2. The overlap of false positive CNV calls using 300 ClinSeq© samples.
